# A Case of Chronic Interstitial Pancreatitis of Uncertain Origin
1Read at the meeting of the Bristol Medico-Chirurgical Society, December 13th, 1911.


**Published:** 1912-03

**Authors:** J. Michell Clarke

**Affiliations:** Professor of Medicine, University of Bristol, and Physician to the Bristol General Hospital


### A CASE OF CHRONIC INTERSTITIAL PANCREATITIS OF UNCERTAIN ORIGIN.[1]

BY

J. MICHELL CLARKE, M.A., M.D. Cantab., F.R.C.P.,

*Professor of Medicine, University of Bristol, and Physician to the Bristol General Hospital.*

THE following case was most obscure from the clinical point of view, and the diagnosis was not made during life. This is not surprising, as according to the last edition of Allbutt's *System of Medicine* "the vast majority of cases of fibrosis of the pancreas are only discovered on the *post-mortem* table." Moreover, in this case

[1] Read at the meeting of the Bristol Medico-Chirurgical Society, December 13th, 1911.

the signs that give most aid in such a diagnosis were absent, as also were the usual causes of the disease. The patient was a teetotaller, and had always lived a very quiet life. There was no history of any severe abdominal illness or injury, or of any affection of the gall-bladder or ducts. There was also no jaundice throughout the illness ; the absence of this symptom materially adds to the difficulty of diagnosis of chronic indurative disease of the pancreas. But perhaps what added to the difficulty more than anything else was the character of the stools : they were dark in colour throughout, and consisted of small scybalous masses. As enemata were mostly used to secure an evacuation, the stools were constantly seen. I think it was the absence of the large, soft, and sometimes pale stools, rich in undigested fat, that commonly characterise disease of the pancreas that diverted one's attention from this organ. It is worth while to bear in mind the lesson of this case, that a fibrosis of the pancreas progressing steadily to a fatal termination may be associated with dark, scybalous evacuations without naked-eye evidence of fat, and the necessity of further investigation of the fæces in the laboratory in a case of similar obscure abdominal disease. I do not attach much importance in the diagnosis to the absence of glycosuria ; when present, it is an aid, but it is frequently absent in pancreatic diseases. The lesion suspected during life was malignant disease, probably of the stomach.

Looking back upon the case, the chief diagnostic features were the severe epigastric pains and attacks of vomiting, not distinctly related to food, and in the absence of any lesion of the gall-bladder or ducts and of enlargement of the liver, the persistent anorexia, the epigastric tenderness, the rapid and profound emaciation, and the great prostration ; but even with the whole history of the case and the *post-mortem* findings in view, it seems doubtful to me whether a positive diagnosis could have been reached, unless an examination of the stools had cleared it up.

Cancer of the pancreas is rarely unattended with jaundice, at any rate during some part of its course.

With regard to the cause of the pancreatic disease, the atrophic condition of the mucous membrane of some parts of the intestine, the distension of the cæcum and thinness of its walls, the very striking alterations in the pancreatic duct, and the evidences of irritation of the abdominal glands, may be taken together as evidences of an infection of the pancreas through its duct from the intestinal tract, most probably of bacterial origin, though the *post-mortem* failed to show the exact nature of the irritant, whether bacterial or bacterio-toxic.

Julia H., aged 44, single, a nurse. Family history good. Seven years ago she was operated upon for strangulated hernia, and since then she has worn a double truss. No other illness and no dyspepsia or other gastric trouble until the last two or three years. She has never had jaundice ; never very strong. For years she had been subject to "piles," as evidenced by attacks of bleeding from the rectum, each of which lasted for a few days. Two severe attacks of this kind occurred in May and June, and since then she had suffered from anæmia. For two or three years past she had complained of pains in the epigastrium, not very severe, worse after food, and of headache.

Her health was much as usual until the middle of July, 1911, when she had a more severe attack of bleeding from the rectum than she had ever had before. On examination by Dr. Pinniger, under whose care she was, nothing but a hemorrhoidal condition of the rectal mucous membrane was found to account for the bleeding. Under appropriate treatment, with rest in bed, the hemorrhage gradually ceased, and did not recur. She was very weak, and on an attempt being made to feed her up she had a sudden very severe attack of epigastric pain, which was not relieved by the vomiting which accompanied it. From this time onwards she suffered from great weakness and prostration, from attacks of faintness, and at frequent intervals from vomiting, with more or less severe pain referred to the region of the epigastrium.

She was admitted to the Hospital towards the end of August, 1911, and I first saw her on my return on September 3rd. During this time she was in bed, extremely weak, anæmic, and with frequent attacks of severe pain in the middle of the epigastrium, at times almost unbearable, from persistent nausea and anorexia, and from attacks of vomiting, which came on suddenly and bore no relation to food. The vomit consisted of the contents of the stomach at the time, and contained no blood.

On September 4th her condition was as follows. She was anæmic with a pale yellow or lemon-coloured tint of skin, somewhat emaciated, extremely weak, and incapable of the least exertion. She could not stand or sit up. Anorexia. Tongue moist and furred. Bowels constipated; the stools consisted of small, hard, dark-coloured scybala; defecation was painful on account of piles. Teeth had been removed some time ago, and she had artificial ones. Pulse 78, regular, weak, small, empty between beats. Catamenia normal.

The thoracic organs were normal, except that the heart's impulse and sounds were weak.

Abdomen not distended, soft, resonant all over, moved normally with respiration. There was no enlargement of the liver or spleen, and the kidneys were not felt. Gall-bladder not felt. The epigastric region was full, resistant, and tender; there was an especially tender spot and perhaps a slight swelling deep in mid-epigastrium, just to right of middle line.

Urine: sp. gr. 1010; no albumin, sugar, or bile; no deposit.

Blood: red cells 4,000,000, hæmoglobin 60 per cent., whites 5,000.

Rectal examination was painful owing to the presence of internal piles; there were no external ones, and no growth was felt. There was some prolapse of rectum.

During the first half of September the pains in the epigastrium continued at frequent intervals. The nausea was relieved for some days by drop doses of tinct. iodi, but then returned. She vomited about every second day. The vomit was acid and never contained any blood. Tests for "occult" blood in vomit and fæces were negative. She took milk diet, and an attempt to give her more solid food only resulted in increase of pain and in vomiting.

The urine varied in specific gravity from 1010–1020, was normal in quantity, occasionally contained a trace of albumin; it never contained bile, and there was no excess of urobilin (spectroscope) nor of aromatic bodies (Jaffé's test). There were no crystals or other deposit. The skin was not jaundiced, the conjunctivæ were white. The stools remained scybalous and dark in colour, and showed to naked eye no excess of fat.

The abdomen remained soft, but there was always excessive tenderness in the epigastrium, a feeling of resistance, and of an indefinite, deep-seated, small swelling midway between ensiform cartilage and umbilicus, just to the right of the middle line.

For about a week at the end of September she improved a little, was less weak, and the pain and vomiting ceased; but on October 4th the pains in the epigastrium returned with increased severity, and attacks of vomiting were frequent and not controlled by the various remedies tried. The bowels were kept open by enemata and mild aperients; they were always constipated, and scybala could occasionally be felt in the colon. She lost 5 lb. in weight from September 4th to 25th.

Blood examination on September 25th gave reds 4,500,000, hæmoglobin 60 per cent., whites 6,800.

Differential count—Polymorphs, 80 per cent.
Small lymphocytes, 8 per cent.
Large ,, 8 per cent.
Transitionals, 4 per cent.
Eosinophils, 0 per cent.

From October 10th onwards, without any change in the symptoms or the appearance of any fresh ones, her condition steadily deteriorated. She was utterly prostrate, dull and apathetic. She lost her voice and emaciated rapidly. The pain and vomiting continued. The heart sounds were extremely feeble. The pulse remained at 110 to 120, was very feeble, and at times could hardly be felt.

The temperature throughout the illness was subnormal, varying from 97° to 98.4°.

On October 19th, after an attack of vomiting, she became cold and collapsed, and died in the early morning.

Throughout the case there were no hemorrhages.

At the *post-mortem*, which was made by Mr. G. Scott Williamson, there were a few slight adhesions over the lungs, which were congested at their base.

*Heart.*—There was an excess of epicardial fat. The heart muscle was paler than normal and striated with white lines; the papillary muscles especially were pale. The aortic valves were thickened, and the commencement of the aorta showed patches of atheroma; mitral segments also thickened with a nodule of atheroma on the aortic one.

*Abdomen.*—There was no ascites.

*Liver.*—Somewhat decreased in size. There were no marked changes; it was rather pale, and on the diaphragmatic surface of the right lobe there were a few pale yellow or mottled patches, the size of a sixpence, with well-defined edges, but not raised above the surface. The section presented a homogeneous appearance. Gall-bladder and ducts, both cystic and common, were normal. No gall-stones. In the mesentery and small omentum were several small areas of fat necrosis.

*Pancreas.*—Weight 9½ oz. Distinctly enlarged, very hard and nodular throughout its whole length. The head felt especially hard. On section it was lobulated with pale areas differentiated from the general structure, and though very hard on cutting, there was no microscopic evidence of fibrosis.

*Spleen.*—Showed enlarged and thickened trabeculæ; the pulp appeared homogeneous, and the Malpighian bodies were not differentiated.

*Kidneys.*—Enlarged, swollen; capsules stripped easily, leaving a pale, smooth surface mottled over with dark areas. The organs showed advanced fatty degeneration, the cortex being pale yellow and soft, and showed no differentiation of its component parts, the vasa recta being only visible at the edge and the medulla hyperæmic. Suprarenals appeared normal.

*Stomach.*—Normal; no sign of malignant disease; pylorus patent.

*Small intestine.*—Mucous membrane somewhat atrophied and smoother than usual. The lymphoid follicles were not prominent.

*Large intestine.*—The cæcum was much dilated and its walls thin. The appendix was normal. The sigmoid flexure and upper part of rectum showed two or three small diverticuli. There were numerous enlarged abdominal glands.

*Microscopic examination.*—In sections stained with pyronin and methyl green the pancreas showed an advanced interstitial cirrhosis of a chronic kind, wide strands of fibrous tissue separating the acini. The connective tissue was not of very old standing, and showed no great tendency to contract; in a few places there were small aggregations of small round cells amongst the fibroid tissues. The cirrhosis was of the interlobular variety, and the newly-formed tissue did not penetrate within the lobules, the mode of spread being by cutting off outlying cells at the periphery of the lobules, which cells then atrophied. The pancreatic cells were normal as regards granules, nuclei, etc., and the intercalary ducts were normal and patent. The islands of Langerhans were well shown by this stain, and were entirely unaffected. In two or three places islets could be seen lying amongst the interlobular connective tissue.

Stained by Scharlach R, the cells of the acini were almost entirely free from fat, but a few contained some fine particles. Within the cells and the lumen of the intercalary ducts, both minute and larger, there was a fairly abundant deposit of mostly small droplets of fat. The chief amount of fat was, however, in the lymph-spaces of the interlobular connective tissue, especially near the periphery of the lobules, and more especially in those parts in and about the aggregations of small round cells, where in many cases there was a large deposit of fat droplets. The fat stained a brownish-red colour. The pancreatic duct showed very striking changes. In longitudinal sections it exhibited a series of constrictions with intermediate swellings, whose cavities were often filled with desquamated cells. At the constrictions the wall of the duct showed dense fibrous tissue, closely packed with round cells, mast-cells and plasma-cells. No micro-organisms were found in the lumen of the duct. The vessels of the pancreas were healthy.

The liver showed very extensive deposit of fat within the liver cells, chiefly in the peripheral, but also in the middle zone of the lobules. In the peripheral zone the fat was in the form of large droplets of the type formerly called " fatty infiltration," but in the middle zone it was in very small particles. There was a large but varying deposit of iron-free pigment granules in the cells at the centre of the lobules around the central vein. The hepatic arteries and ducts were normal ; there was no increase of connective tissue in the liver, and it was not congested.

The spleen showed no change of importance, the pulp-vessels were very full of blood, and the trabeculæ large and prominent in sections.

In the kidney the medulla showed extreme congestion of the vessels. In the cortex the vessels were not congested, and the glomeruli were normal ; the convoluted tubules in parts showed epithelium which was swollen and granular, probably partly due to cloudy swelling and partly to early fatty degeneration. In other areas the normal epithelium of the tubules had disappeared, and they were lined with a single row of flattened cells.

The enlarged abdominal glands, which were numerous, were in a state of extreme hyperplasia, with an unusual number of polymorphonuclear leucocytes and of endothelial cells with cell inclusions.

I must not conclude without expressing my best thanks to the pathologist to the Hospital, Mr. G. Scott Williamson, for his very excellent sections.

